# 4D Printing: A Review on Recent Progresses

**DOI:** 10.3390/mi11090796

**Published:** 2020-08-22

**Authors:** Honghui Chu, Wenguang Yang, Lujing Sun, Shuxiang Cai, Rendi Yang, Wenfeng Liang, Haibo Yu, Lianqing Liu

**Affiliations:** 1 School of Electromechanical and Automotive Engineering, Yantai University, Yantai 264005, China; chuhonghui1993@163.com (H.C.); sunlujingslj@163.com (L.S.); yangrd@ytu.edu.cn (R.Y.); 2 School of Mechanical Engineering, Shenyang Jianzhu University, Shenyang 110016, China; liangwf@sjzu.edu.cn; 3 State Key Laboratory of Robotics, Shenyang Institute of Automation, Chinese Academy of Sciences, Shenyang 110016, China; yuhaibo@sia.cn (H.Y.); lqliu@sia.cn (L.L.)

**Keywords:** four-dimensional (4D) printing, additive manufacturing, smart materials, shape memory polymer

## Abstract

Since the late 1980s, additive manufacturing (AM), commonly known as three-dimensional (3D) printing, has been gradually popularized. However, the microstructures fabricated using 3D printing is static. To overcome this challenge, four-dimensional (4D) printing which defined as fabricating a complex spontaneous structure that changes with time respond in an intended manner to external stimuli. 4D printing originates in 3D printing, but beyond 3D printing. Although 4D printing is mainly based on 3D printing and become an branch of additive manufacturing, the fabricated objects are no longer static and can be transformed into complex structures by changing the size, shape, property and functionality under external stimuli, which makes 3D printing alive. Herein, recent major progresses in 4D printing are reviewed, including AM technologies for 4D printing, stimulation method, materials and applications. In addition, the current challenges and future prospects of 4D printing were highlighted.

## 1. Introduction

In 1986, Chuck Hull proposed that three-dimensional (3D) systems applied for a technology of stereolithography (SLA), which attracted the world’s attention and to some extent represented the origin of a 3D printing technology [1]. Since the late 1980s, additive manufacturing (AM), often referred to as 3D printing or rapid prototyping, has been gradually popularized [2,3,4]. Currently, additive manufacturing for four-dimensional (4D) printing is mainly divided into two categories: Extrusion-based methods [5], and vat photopolymerization methods [2,6]. 3D printing has been widely used in biomedicine, polymer science, space science, and other fields by virtue of its rapid prototyping of 3D products with complex shapes [7,8,9,10,11]. However, he microstructures fabricated using 3D printing is static. On the other hand, while 3D printing has made great breakthroughs in all aspects, its limitation lies in the layer-by-layer printing speed. On account of some of the shortcomings mentioned above, 3D printing still cannot completely replace traditional manufacturing [12]. In the industry, 3D printing technology is mainly used for sample manufacturing in the early stage of the development of new products, especially those with a complex structure.

3D microstructures made of smart materials, relying on the functions of these materials, can evolve over time in a predetermined manner. This has given rise to a new term, i.e., “4D printing” [13]. In 2013, professor Tibbits first proposed the concept of 4D printing [14]. In a TED talk in 2013, he defined 4D printing as a new design of a complex spontaneous structure that changes with time due to the interaction of environment, marking the emergence of the concept of 4D printing. 4D printing was originally defined with the formula of “4D printing = 3D printing + time”, which refers to changes in the shape, structure, or function of 3D printing over time [15,16,17]. It is a purposeful evolution of 3D printing structure in shape, structure, and function, intended to effectively realize self-assembly, deformation, and self-repair. Zhong et al. defined 4D printing as the AM process that integrates smart materials into the initial form of printed materials for 3D printed structures/components [18]. With the continuous development of research and technology, the definition of 4D printing will be more comprehensive. 3D printing is “pre-modeling + printing of the finished product”, while the idea of 4D printing is to embed the design of the product into a flexible smart material based on 3D printing. Therefore, microstructures can be deformed according to the pre-designed track under specific time and activation conditions. Currently, 4D printing can create many objects that 3D printing cannot, and the color [19,20], volume [21], and shape [22] of these objects can change with environmental conditions and stimuli, such as water and temperature [14]. Compared to traditional manufacturing methods, 4D printing is advantageous in terms of material adaptability, which facilitates the precise configuration of material responsiveness. 4D printing has been applied to some fileds successfully. For instance, bioprinting is an emerging technology, whose greatest advantage lies in its ability to create 3D structures of living things, such as tissues, organs, nutrients, and cells [23,24,25]. 4D bioprinting technology, which can be widely used in regenerative medicine, materials science, chemistry, and computer science, is emerging as the next-generation bioprinting technology [26,27,28,29]. The main advantages of 4D bioprinting are that fabricated bio-structures can alter their functionalities [30].

In this review, we first introduce additive manufacturing techniques for 4D printing, describe the advantages and disadvantages of the objects printed using additive manufacturing, and put forward the concept of 4D printing. Then some methods of implementing 4D printing are presented. The next part classifies and summarizes some stimuli that 4D printing can respond to. Next, the smart materials used in 4D printing, including shape memory polymers (SMPs) and hydrogels, are introduced. Finally, some applications of 4D printing and the trend of 4D printing and the perspectives for this exciting new field are highlighted.

## 2. Additive Manufacturing (AM) Technologies for Four-Dimensional (4D) Printing

Additive manufacturing technologies used for 4D printing can be classified into several categories based on the mode of materials or ink deposition: Extrusion-based methods including fusion deposition modeling (FDM), direct ink writing (DIW) and inkjet, as well as vat photopolymerization methods including stereolithography (SLA) and digital light processing (DLP).

### 2.1. Extrusion-Based Methods

#### 2.1.1. Fusion Deposition Modeling (FDM)

**Figure 1:**
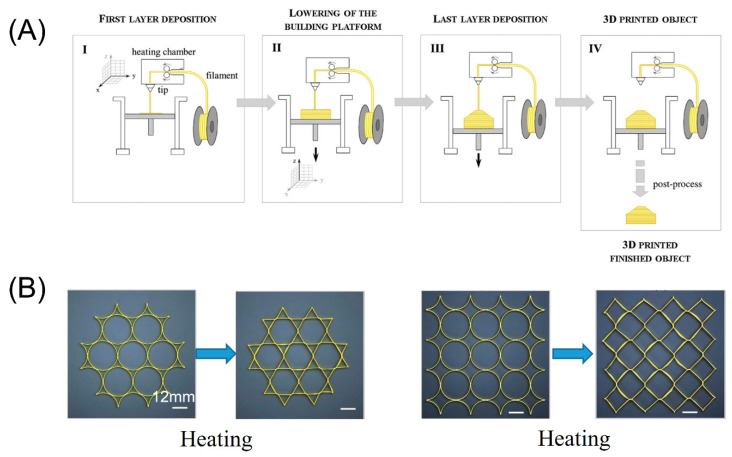
(**A**) The entire process of fusion deposition modeling (FDM). (Reproduced with permission from [47]) (**B**) polylactic acid (PLA)-based circular rings and square lattices printed using fusion deposition modeling (FDM) were transformed into hexagons and quadrangles when heated to 90 °C. (Reproduced with permission from [38]).

FDM, also called fused filament fabrication (FFF), is based on the working principle of extrusion [31,32]. FDM is an easy-to-use, low-cost and fast-printing technique due to the non-requirement of chemical reaction and low price of equipment and materials. Under FDM, solid filaments are melted in a heated nozzle, forming a 3D object on the base of the printer in a line-by-line and layer-by-layer manner (Figure 1A). The printing materials should meet the requirements that can flow after being melted and solidify. Various polymers including polylactic acid (PLA), acrylonitrile butadiene styrene (ABS), polycarbonate (PC), polycaprolactone (PCL), polyethylene terephthalate (PET), and polyphenylsulfone (PPSF), are the ideal materials for this application. The mechanical properties are an important parameter of FDM printed general polymer parts and depend on filament bonding influenced by slicing parameters, building orientation and temperature conditions [33]. Wach et al. demonstrated that the mechanical properties of FDM-PLA parts can be enhanced by thermal annealing [34]. Furthermore, the mechanical properties of polymers could be reinforced with discontinuous fibers. Lee et al. fabricated long and discontinuous natural fiber to enhance the mechanical properties of polypropylene [35]. The nozzle is driven by the motor, and moves on the two horizontal printing axes of x axis and y axis. Due to the different diameters of nozzles, the resolution of FDM is limited, generally within the range of 100–200 μm [36,37]. When using the FDM method, the most rational polymer is thermoplastics which can be melted quickly after heating and can solidify these excellent properties after cooling. In recent years, FDM has aroused the interest of many researchers. Zhang et al. printed PLA strips which can achieve pattern transformation during thermal stimuli (Figure 1B) [38]. PLA-based circular rings and square lattices were printed onto the platform using FDM and solidified by cooling below its *Tg*. These lattices could be transformed into hexagons and quadrangles when heated to 90 °C due to the internal stress accumulated in the material. In general, the interface adhesion between two different materials is poor because of the different printing temperatures of different thermoplastics, so it is difficult to print multiple materials using the FDM method [39]. Tian et al. proposed a continuous fiber-reinforced thermoplastic composite (CFRTC) based on PLA and continuous carbon fiber as a new approach to 3D FDM printing manufacturing [40]. CFRTC has attracted extensive attention from researchers, mainly owing to its excellent mechanical properties, low weight, and recyclability. Wang et al. focused on the process of recycling and remanufacturing printed matter, and inspected and demonstrated the research method they proposed. The main factor affecting the performance of this method is the proper setting of temperature and pressure, which leads to differences in the mechanical properties of the material. Tian et al. conducted in-depth research on the interface, quality, and performance of printed objects [41]. In a material with a fiber concentration of 27%, the ideal temperature of the nozzle heater is 200–230 °C, the maximum bending stress is 335 MPa, and the bending modulus is 30 GPa. It is difficult to assure the uniformity of fabricated structures in the vertical direction when using FDM [38].In addition, due to the fabricating process, the step-structure will inevitably be formed on the surface. Le Duigo et al. utilized FDM to fabricate continuous flax fibre/PLA reinforced biocomposites for structural applications [42]. This research group also printed wood fibre biocomposites and the porosity induced by FDM could be tuned [43]. Similarly, Correa et al. presented new methods for designing hygroscopic wood transformations and 3D printing custom wood grain structures to promote tunable self-transformation [44]. Pezzulla et al. fabricated two-dimensional 2D sheets using FDM which can be transformed into 3D shapes by preparing geometric composite structures that deform by residual swelling [45]. Goo et al. programmed FDM printing paths intentionally to impose bidirectional anisotropy and a unique thermal deformation in response to a thermal stimulus was generated [46].

FDM is widely employed to develop the 3D printer, however, this techniques still has various drawbacks to overcome. As we mentioned above, a resolution of x–y and z-axis was limited by the nozzle dimensions and fabricating geometrical complexity of the part seems difficult. Furthermore, the accuracy of the printing process depends on a complex mix of factors including flow rate, material properties, heat transfer dynamics and deformation after or during the process. Printer filaments were melted above the melting temperature, which may cause invalidation of components in the filaments, thus greatly limiting their application in requiring a long period of stability.

#### 2.1.2. Direct Ink Writing (DIW)

**Figure 2:**
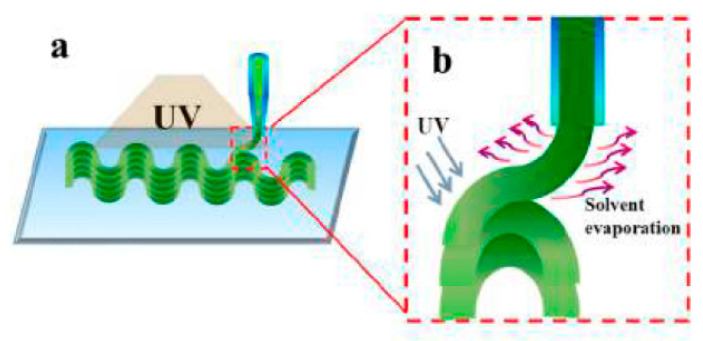
Schematic illustration of the direct ink writing (DIW) which can be used for four-dimensional (4D) printing. (**a**) Ultraviolet (UV) crosslinked ink was extruded from a micro-nozzl; (**b**) the ink will be cured under UV exposure (reproduced with permission from [39]).

DIW is one of the widely used AM processes based on the principle of extrusion constructing microstructures layer-by-layer by using a computer-controlled robot to move the dispenser filled with printed ink [48,49,50]. It can provide an excellent interface combination and adjustable mechanical properties. So far, various types of materials including metal particles, polymers and ceramics can be printed successfully using DIW. According to this process, a viscous liquid ink that can be cured later is deposited, forming a 3D object line-by-line and layer-by-layer, as in the case of FDM. The necessary conditions for the extrusion process include proper shear thinning [51] and other rheological properties [52]. In the extrusion process, with the increase of shear stress, the viscosity of the ink will decrease gradually, and the ink can even flow freely such as liquid and will not generate excessively high pressure inside. In addition, there are several ways to keep the printed structure stable. Wei et al. used DIW to print a 4D architecture based on UV cross-linked PLA (Figure 2) [39]. This system can be thermally driven or remotely driven because of the addition of Fe_3_O_4_ nanoparticles in the system. First the ink is extruded through the micro-nozzles of the syringe. After that, the solvent evaporates, and UV light is used to irradiate the cross-linked polymer. In this way, the ink experiences a quick transition from liquid to solid state, and the desired filament structure is obtained. Zolfagharian et al. 4D printed photoresponsive structures consisted of shape memory polystyrene, chitosan, and carbon black [53]. Polystyrene was firstly printed above *Tg* and cooled into the shape below *Tg* (*Tg* ≈ 102 °C). The adhesion and transparency can be regulated by addition of chitosan and carbon black. Inspired by plant architectures, Gladman et al. designed a 4D flower-like structure prepared using this method, which is different in that its structure is water responsive [17]. Controlled anisotropic alignment of cellulose fibrils were printed and these fibrils in different directions endowed flower microstructures that showed different swelling ratios in water, thus triggering biomimetic shape transformation. Chen et al. 4D fabricated microstructures with graded multi-materials via photomask-assisted DIW with a two-stage curing method [54]. The crosslinked networks of photocurable resin and shape memory epoxy interlocked physically and two graded structures consisting of three levels were formed using this method. In recent years, it has been reported that the high resolution of DIW can be achieved through micro-nozzles [55]. On the other hand, DIW can be used in multi-material printing, provided that the two polymer resins are compatible. Wei et al. printed a spiral and cubic scaffold with highly conductive multimaterial composites and connected into a circuit [56]. The light emitting diode (LED) can work under 2.5 V due to the high electric conductivity of this scaffold.

Compared with other methods, the DIW technique shows superiority due to the multiple choice of materials, low material consumption, open source of controlling machines and feasibility for multi-material printing. However, the cost of fabricating system is high and the build volume is small. The viscosity of ink needs to be carefully regulated because it is required to possess a specific rheological performance.

#### 2.1.3. Inkjet

**Figure 3:**
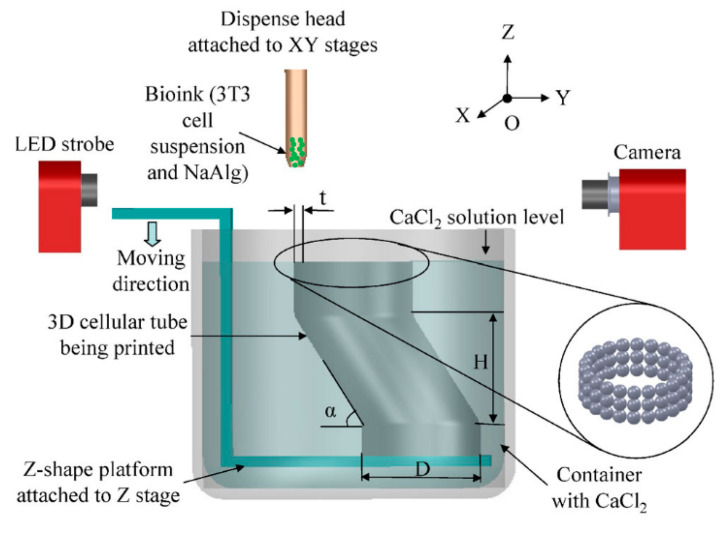
Schematic illustration of the platform-assisted three-dimensional (3D) inkjet bioprinting system used for 3D zigzag cellular tubes fabrication (Reproduced with permission from [60]).

Another popular printing method is inkjet printing [57,58,59]. Its working principle is that single and multiple nozzles can work at the same time, and that different light-curable liquid resins can be sprayed on the printing platform to form a layer, which is finally used for light curing (Figure 3). In this manner, components composed of multiple materials can be manufactured under a relatively high resolution. However, compared with single nozzles printing, the typical planar resolution of an inkjet printer drops rapidly from 30 μm–40 μm to 200 μm–400 μm for multi-material printing. Actually, inkjet printing is a rather fast and cheap technique and a wide range of materials type are available.

### 2.2. Vat Photopolymerization Methods

#### 2.2.1. Stereolithography (SLA)

**Figure 4:**
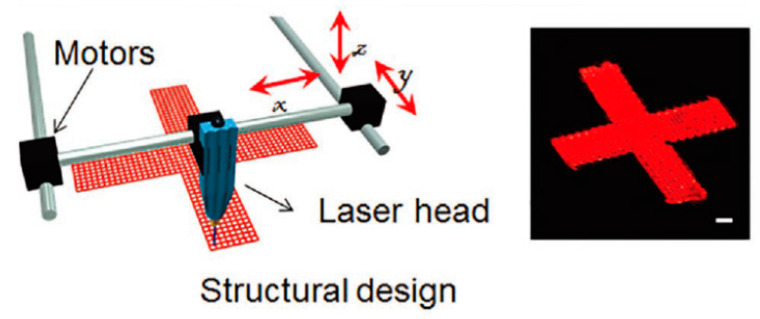
Schematic illustration of the stereolithographic (SL) printing which can generate parts with high accuracy and has been widely used (Reproduced with permission from [68]).

SLA was developed by Charles Hull in the mid-1980s, and is currently one of the most widely used rapid prototypes [6,61]. Generally, a liquid resin was polymerized and cross-linked into a solidified polymer induced by light sources in the SLA system. SLA is characterized by high spatial resolution and high fabrication speed [62]. The light source can bring a change in energy, which further leads to the curing reaction of the material, thus obtaining the desired solid structure. In this regard, gamma rays, X-rays, electron beams, ultraviolet (UV) rays, and visible light are commonly used radiation means for curing. Commercial radiation mainly includes UV and visible light. As shown in Figure 4, the building surface is placed upside down on a relatively shallow resin vat, and there is a transparent window at the bottom of the resin vat. The light shines on the building surface through the transparent window, and the thickness of each layer printed mainly depends on the distance between the building surface and the transparent window. Once a layer is completely printed, the building surface will move up, and then the next layer will be printed, etc., until the entire structure is completely printed. In this type of device, since the light source is above the build surface, the precursor can be selectively cured. Choong et al. used SLA to print buckminsterfullerene with a tert-Butyl acrylate -co-di(ethylene glycol) diacrylate (tBA-co-DEGDA) network based on the dual-component phase switching mechanism [63]. Theses buckminsterfullerene could be unfolded after printing at the temperature of 25 °C and recovering its original bucky-ball shape by soaking at 65 °C of water, which exhibits a shape memory behavior. Zarek et al. used the aforementioned technology, namely, the inverted SLA technology, to manufacture the shape memory structure of PCL [64]. The uniqueness with this technology is that the platform is sunk into a methacrylated PCL oligomer melting vat to print out the PCL sheet. Zhao et al. synthesized a type of UV-curing polyurethane prepolymer and this polymer was used to fabricate objects using SLA [65]. The recovery angle of microstructures is inextricably linked to temperatures in water bath. Lu et al. developed a magnetic-assisted stereolithography to fabricate material intelligence for various new applications [66].

SLA can print parts with more complicated geometric shapes [67], and with a somewhat better surface finish than that of traditional machined parts. With the continuous advancement of technology, functional materials for SLA technology have been developed. For example, smart materials were synthesized for SLA and the printed microstructures with high shape fixity and excellent shape memory performance [65].

#### 2.2.2. Digital Light Processing (DLP)

**Figure 5:**
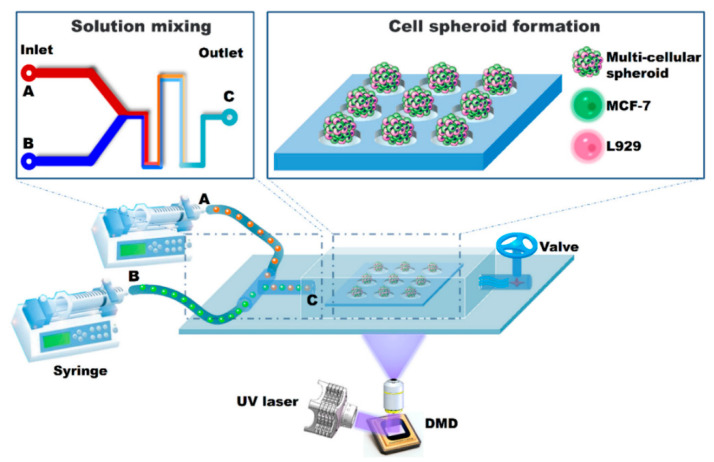
A mask-free method of multicellular heterospheroids arrays formation using digital micromirror device (DMD)-based hydrogel fabricating system. The solution mixing part consists of two inlet (A) and (B), and one outlet (C). (Reproduced with permission from [70]).

Compared with traditional SLA technology, DLP technology can cure the entire pattern in a layer in a single exposure, thereby improving efficiency (Figure 5) [69,70,71]. Zhao et al. used the DLP method to print environment-responsive self-folding origami structures based on polyethylene glycol diacrylate (PEGDA) [72]. By adjusting the intensity of light, thin polymers with different degrees of crosslinking can be obtained. Ge et al. used a high-resolution DLP in manufacturing multi-layer shape memory objects composed of various methacrylate copolymers, which is called projection micro stereolithography (PμSL) [73]. Invernizzi et al. printed objects using the PCL/ ureido-pyrimidinone (UPy)-based polymer by DLP and these materials showed good shape memory and self-healing properties which can act as soft actuators [74]. Li et al. developed a new shape memory polyimide ink with high mechanical strength and low contraction and printed a self-folding box and stimuli-response gripper [75]. Devillard et al. used the DLP-based 3D printing technology to generate a biomimetic construct of vascularized alveolar bone [76].

DLP, as an emerging rapid printing technology, has great potentials in the future of 4D printing [77,78,79]. It is one of the additive manufacturing technologies that is closest to the production level, and it can achieve high resolution on both micro and nano scales through optical lens systems. Compared with SLA, this technique possesses higher print speed. In addition, with the continuous development of technology and the advancement of materials science, DLP has been developed to combine with a two-stage curing method to manufacture engineering polymers.

## 3. Stimulation Method of four-dimensional (4D) Printing

The shape and function of fabricated structures can be changed according to one or more stimuli. There are two categories of stimuli, i.e., external stimuli and internal stimuli. External stimuli mainly include water/humidity, temperature, light, electric field, and magnetic field, while the main internal stimulus is the cell traction force. The theory, advantages and disadvantages of each method were shown in Table 1.

### 3.1. Water/Humidity Stimuli

**Figure 6:**
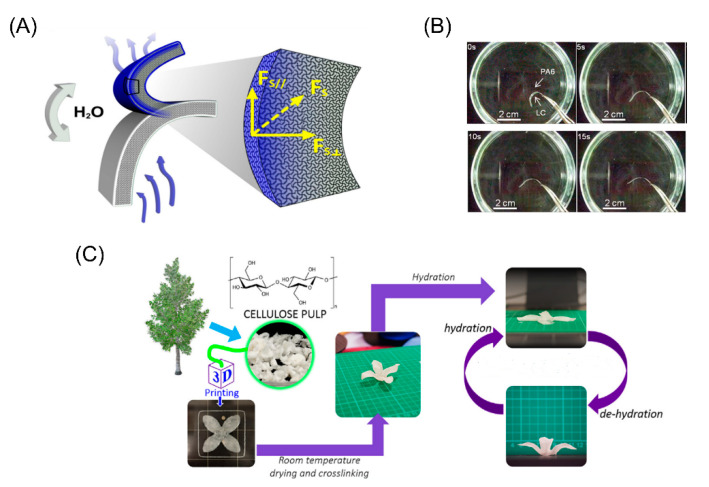
(**A**) Schematic illustration for the water-responsive bending of cellulose stearoyl esters (CSE)0.3 film and the blue layer indicates the film with absorbed water and the white layers indicate the film without water (Reproduced with permission from [98]). (**B**) The fabricated structure will be deformed and shows self-expansion/shrinkage under different environment (Reproduced with permission from [81]). (**C**) A stimuli responsive cellulosic pulphydrogel composite ink was developed and the petal architecture fabricated using this ink could deploy to flat configuration upon hydration and recover from drying (Reproduced with permission from [100]).

Water and humidity were first used as stimuli in 4D printing [17,80,96,97]. Materials sensitive to water or humidity are of great interest because of their ubiquitous irritation and wide application. By using water as an external stimulus, the structure can be deformed underwater and restored to its original shape after drying. However, the degree of expansion/contraction of the humidity-sensitive material should be precisely controlled during the transition to maintain the integrity of the printed structure. Zhang et al. developed a material sensitive to water by modifying cellulose with stearyl [98]. A film was made using the material and once the film was placed in an environment with a water gradient, bend deformation would occur due to the uneven absorption of water (Figure 6A). Lewis et al. mixed cellulosic fibrils with acrylamide as a composite ink for printing the original flat structures and the anisotropic swelling behavior can be regulated by the alignment of cellulose fibrils along printing pathways when immersed in water. The results indicated that the combination of materials and geometry can be controlled in space and time [17]. Villar et al. demonstrated a two-layer osmotic mechanism with a lipid interface combining two picoliters of water droplets at two different osmotic pressures [99]. Droplets under a high osmotic pressure will swell, while those under a low osmotic pressure will shrink until they reach the same osmotic pressure. As shown in Figure 6C, Mulakkal et al. developed a stimuli responsive cellulosic pulphydrogel composite ink and the petal architecture fabricated using this ink could deploy to a flat configuration upon hydration and recover from drying.

### 3.2. Temperature Stimuli

Temperature is one of the most commonly used shape-shifting stimuli in polymer-based materials [101,102,103]. Ge et al. printed an SMP flower which could bloom when heated [73]. The technology is also used to make smart grippers that do not require assembly or electromechanical components. The latest discovery by Bodaghi et al. shows that SMP structures can be preprogrammed by taking full advantage of the heating process in FDM printers [104]. Hu et al. demonstrated a graphene-based bipiezoelectric structure that expands into a plate when heated and rolls back into a cylinder when cooled [105].

**Figure 7:**
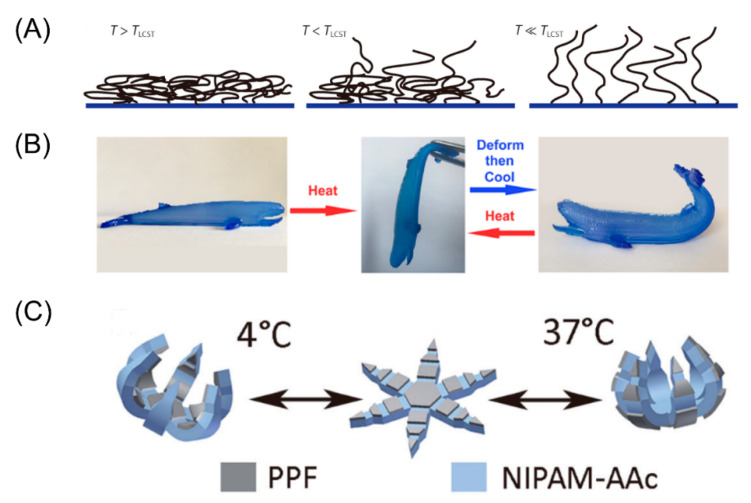
(**A**) Schematic illustration of the chain conformations of Poly(N-isopropylacrylamide) (PNIPAM) corresponding to the volume fraction profiles under various temperatures (Reproduced with permission from [109]). (**B**) A whale like hydrogel printed by 4D ink will deform when heated and cooled. (Reproduced with permission from [110]). (**C**) Theragrippers fabricated using PPF and pNIPAM-AAc. The grippers can reversibly open and close around body temperature because of the thermal responsiveness of the pNIPAM-AAc. (reproduced from [111]).

Wang et al. established a phenomenological model, and introduced the concept of phase evolution to describe the glass transition behavior of SMP [106]. In a typical shape memory cycle, the SMP sample first deforms from its original shape at a temperature higher than its transition temperature, and then cools to a lower temperature under the condition of maintaining external constraints. Fiber-based glass-polymers exhibit a shape memory effect (SME) when heated above their glass transition temperature (*Tg*). Figure 7A shows the typical deformation process of SMPs under the stimulus of temperature. The printing strip is initially flat in shape, and when heated above the temperature Tg, the shape memory material behavesas a rubber, and an external force is applied to the end of the band. The strip is then cooled to a lower temperature, where the shape memory material appears as a rigid solid. Due to the uneven thermal stress inside it, the strip is bent [107]. If reheated, the shape memory material will become elastic again, and the strip will eventually return to its original flat state. Using a polystyrene film sensitive to thermal stimuli, Deng et al. designed a mechanism to obtain self-folding 3D circuits based on DIW [108]. Resin was used for one side of the film as a constraint layer, while the other side was left empty. As a result, by raising the temperature, the empty side was folded on the hinge.

### 3.3. Light Stimuli

Light is a common stimulus that regulates the polymer shape through remote induction. The polymer shape can be changed using a light trigger with different wavelengths. Since it doesn’t bring any damage to the cells such as increasing the temperature of the material, this stimulus can be used in biomedicine and drug delivery in vivo. For example, Luo et al. confirmed that the shape deformation of alginate/polydopamine (PDA)-based scaffold was induced by near infrared ray (NIR). At room temperature, the alginic acid scaffold, which has been approved by FDA and has a good photothermal effect, folds slowly when dehydrated. It can quickly convert the absorbed light into heat, thereby accelerating the dehydration and deformation of the alginic acid scaffold. The bending process of the alginic acid/PDA bilayer could be controlled by the power and exposure time of light. What’s more, it can be used to fabricate stent with well controlled shape change. This method is also widely used in the field of 4D bioprinting, especially in the manufacture of self-folding 4D cell laden structures.

**Figure 8:**
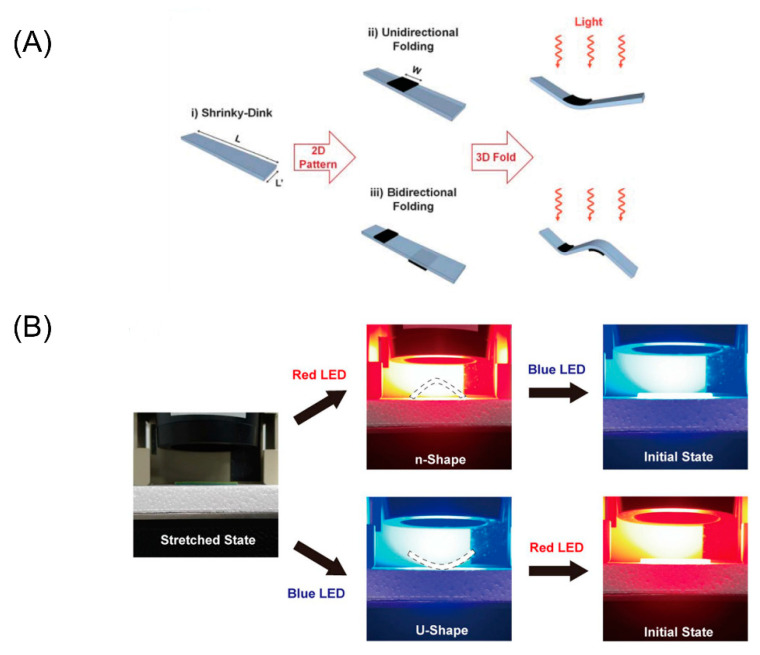
(**A**) The compositionally homogenous sheet of the shape memory polymer will be changed into a hinging response triggered by an inexpensive infrared (IR) light (Reproduced with permission from [114]). (**B**) The multicolor sample bends to a u-shape illuminated by blue light and bends to a n-shape under red illumination. (Reproduced with permission from [113]).

Unlike temperature and moisture, light is an indirect stimulus. It has been regarded as an effective activated 4D printing technology, thanks to its rich source of energy and wireless and controllable properties. Kuksenok et al. used light as a trigger for deformation in a very different way [112]. Parts of polymer gel blocks permeated by a certain number of light-responsive chromophores, could swell only when exposed to light. In addition, the versatility of light as a stimulus is reflected in the printing patterns. Gradient cross-linking at depth can be achieved by projecting UV light on the liquid resin, where anisotropy helps bend the 4D printed structure [81]. As an external stimulus intended to change the color of printed objects, light is advantageous because it can perform high-resolution control in space and time. Jeong et al. demonstrated the multicolor 4D printing of SMPs [113]. By using color-dependent selective light absorption and heating in multicolor SMP composites, they achieved a remote drive with light. The thermo-mechanical programming structure will bend into an n-shape under red lighting. After bending, the structure can return to its original fat state under blue lighting (Figure 8B).

### 3.4. Electric Field Stimuli

**Figure 9:**
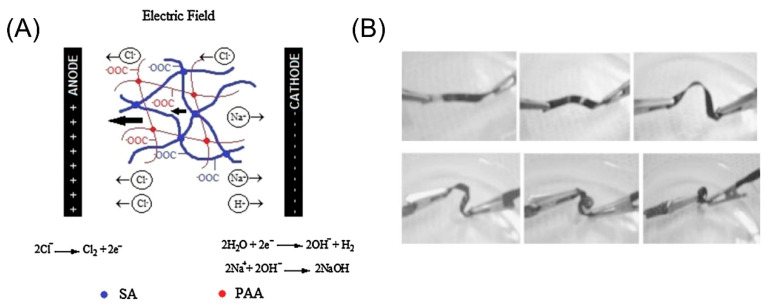
(**A**) The bending of SA-AA-GA film induced by an external electric field (reproduced from [116]). (**B**) The photo series shows the electromagnetic induction shape memory effect of the sample (Reproduced with permission from [120]).

Similar to light, the electric field can also be used as a stimulus in remote control. When used as a stimulus, the electric field produces a resistive drive to fill an SMP with a conductive filler [115,116]. As shown in Figure 9, the photo series shows the electromagnetic induction shape memory effect of the sample. Under the action of an electric field, a single CNT can be polarized by the electrons, and aligned along the direction of the electric field. By applying an alternating current of 300 kHz, it can restore its original straight shape. Miriyev et al. demonstrated a soft, printed artificial muscle made from a mixture of silicone elastomer and ethanol [117]. When an electric field is applied, heat is generated through resistance, causing the ethanol to evaporate. This phase shift from liquid to gas greatly increases the volume of the ethanol, thus expanding the entire matrix [118]. Okuzaki et al. fabricated an origami miniature robot using the PPy membrane. Additionally, this robot has a special geometry on its feet that makes it less resistant to moving forward [119]. When placed in an electric field, the voltage causes the head to move forward by absorbing water, and when the lack of voltage causes desorption, the tail rises.

### 3.5. Magnetic Field Stimuli

**Figure 10:**
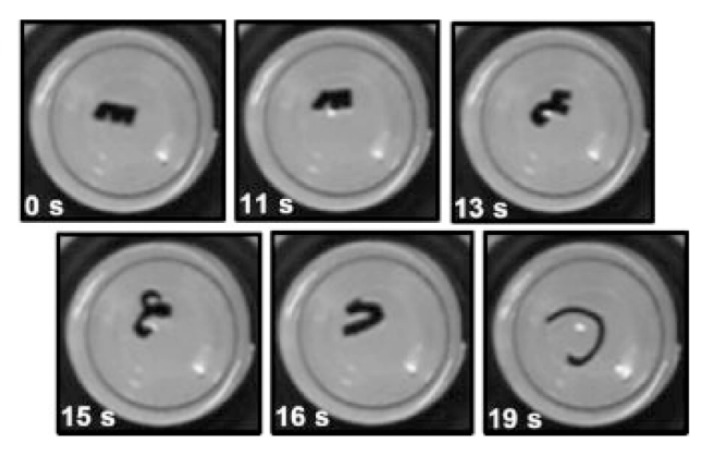
The photo series demonstrating the shape memory polymer containing magnetic nanoparticles transition induced electromagnetic field generated by the topside of the induction coil (Reproduced with permission from [123]).

Magnetically induced shape recovery can be achieved by doping SMP with magnetic nanoparticles (such as Fe_2_O_3_ and Fe_3_O_4_) [91]. Breger et al. combined magnetic nanoparticles into a micro-clamp printed by hydrogel, and achieved remote control by applying a magnetic field [121]. Mohr et al. studied magnetically induced thermoplastic SMP composites filled with Fe_2_O_3_ nanoparticles [122]. The shape recovery of SMP composites can be induced by heating in an alternating magnetic field. The shape memory effect of magnetic induction is exemplified in Figure 10. In addition, Schmidt added surface-modified superparamagnetic nanoparticles (Fe_3_O_4_, diameter: 11nm) to the SMP matrix [123]. Adding Fe_3_O_4_ nanoparticles to the aforementioned PLA printing process not only improves the mechanical properties and shape recovery of the material, but also introduces a magnetic response to the 4D printed structure. Chen et al. fabricated a magnetic hydrogel octopus using AAM-carbomer ink mixed with ferromagnetic nanoparticles, which can be driven remotely by a magnetic field and can move freely in a petri dish [124].

### 3.6. Cell Traction Force Stimuli

**Figure 11:**
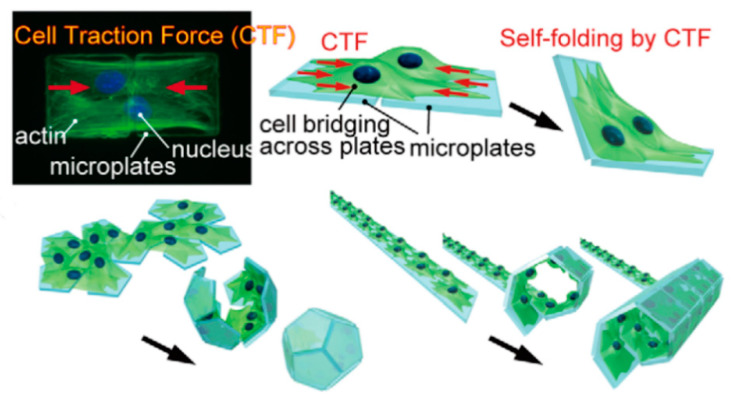
Conceptual illustration of self-folding of three-dimensional cell laden microstructures driven by cell traction force (Reproduced with permission from [128]).

Traction force is generated by the cells attached to the substrate. In biology, cell traction plays an important role in many processes such as cell migration, proliferation, and differentiation [125,126,127]. As shown in Figure 11, Takeuchi et al. made a self-folding structure based on cell traction force. The material used for cell culture here should have sufficient flexibility, and be able to maintain the state of cell adhesion under the action of traction. Due to the traction force of the cell, the flexible joint will be deformed, causing the flat panel to fold. When the microplate is blocked by cells, the folding process is terminated. Therefore, the approximate folding angle can be determined by the number of cells on the microplate. The size of the folding angle is determined by the thickness and width of the flexible joint, and has a certain relationship with the thickness of the microplate itself. One advantage of this method lies in that the folding behavior is induced by the cell without external force, which features a high biological compatibility. However, cell traction is small and hard to control. In addition, the design of structures is crucially important.

## 4. Material System

Currently, materials widely used in 4D printing are shape memory polymers (SMPs) and hydrogels. The main difference between these two types of materials is that changes in SMPs can be programmed after printing.

### 4.1. Shape Memory Polymers (SMPs)

Since the discovery of SMPs, they have attracted the attention of many researchers [129,130]. SMPs shows high stiffness and rapid response to stimulation, which can produce large recoverable deformation after external stimuli (e.g., joule heat, light, magnetism, or water) [131]. In 1941, Vernons first mentioned SMPs, and invented a single synthetic resin with two different flexible physical structures [132]. An SMP must consist of two segments, one highly elastic and the other capable of reducing its stiffness under certain stimuli. The latter can be a molecular switch or a stimulus sensitive domain. After a particular stimulus, a switch/transform is triggered and the strain energy stored in the temporary shape is released, resulting in shape recovery. Through a large number of literature studies, it has been found that most materials printed in 4D have significant shape memory capabilities [133,134]. For example, polylactic acid (PLA), acrylonitrile butadiene styrene (ABS), and polyvinyl alcohol (PVA) all have the ability to change shape when triggered by external conditions [135].

To date, SMP research activities have been carried out in more than 60 research institutes or companies worldwide. In recent years, a variety of polymers with shape memory effects have been synthesized, and some unique properties developed. Based on the shape memory effect, some new multifunctional SMP or nano SMP composites are also proposed [136]. The programmability of temporary shape distinguishes them from other deformed materials. They have potential applications in aerospace, biomedical/flexible electronics, and other fields [137]. In theory, through this procedural design process, an original shape can be fixed into an infinite number of temporary shapes through different deformation processes. SMPs are also stimulus-responsive materials [63,64]. At the same time, according to different shape memory mechanisms, SMP objects also have multiple shape memory effects and reversible shape memory effects, which can memorize multiple shapes and reversible shapes. 4D printing SMPs can not only carry out simple shape changes, but also realize self-deformation, self-assembly, self-repair, and other functions by presetting a deformation scheme (including target shape, attribute, function, etc.). SMPs can be divided into three categories: thermal response type, light response type, and chemical response type.

#### 4.1.1. Thermally Induced SMP

**Figure 12:**
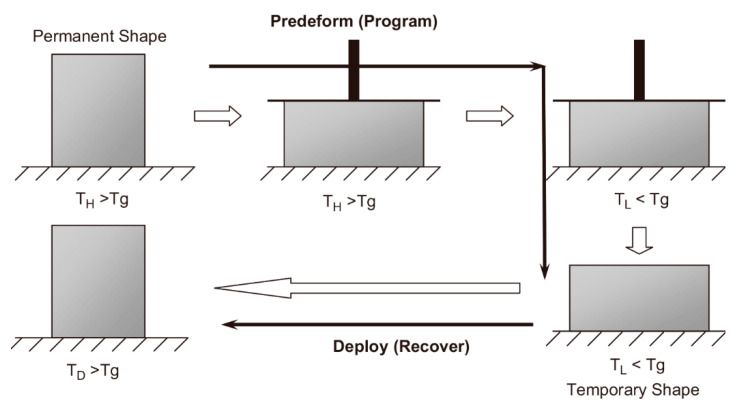
A typical thermo-mechanical loading/unloading cycle in a shape memory polymers (SMP) application (Reproduced with permission from [138]).

The shape memory function of thermally induced SMP mainly comes from the incompletely compatible two phases in the material, that is, the stationary phase and the reversible phase [138]. The function of the stationary phase is to remember and restore the original shape, while the reversible phase ensures that the molded product can change shape. According to the structural characteristics of the stationary phase, the SMP can be divided into two categories: Thermosetting and thermoplastic [139,140,141]. Thermosetting SMP mixes the polymer with the crosslinking agent after heating up to the melting point (tm), and then engages in a cross-linking reaction in the mold to determine the initial shape (Figure 12). After cooling and crystallization, the initial state is obtained. When the temperature rises above tm, the reversible phase melts and softens, making it into any shape under the action of external forces. The external forces are kept and the chains cooled and fixed, so that the molecular chains tend to freeze along the direction of external forces and become morphed. When the temperature rises above tm, the reversible phase molecular chain will naturally curl under the action of entropy elasticity until reaching the thermodynamic equilibrium state, and then shape recovery will occur and the shape will be remembered once.

#### 4.1.2. Photochromic SMP

Photochromic SMP introduced some appropriate photochromic chromophore group (PCG) and when it is exposed to light (usually UV light), the PCG isomerization reaction is going to happen and the molecular chain of molecules will significantly change [142,143]. Furthermore, PCG could produce a reversible isomerization reaction and a molecular chain could form the corresponding recovery. Therefore, the material has the ability to restore the original, but this restoration process is slow. The recovery process can be accelerated by heating or shining light at other wavelengths (usually visible light) after the illumination stops.

#### 4.1.3. Chemical Induction SMP

Chemical induction SMPs are polymers that undergo deformation and recovery under the action of chemicals [144,145]. The usual methods of chemical induction include pH change, equilibrium ion displacement, etc. The mechanism of polymer shape memory is different depending on the stimulus. For example, the stimulus method of pH value change is to soak the polymer in a hydrochloric acid solution, and the mutual exclusion between hydrogen ions will expand the molecular chain segment. When the equivalent NaOH solution is added to the system, the acid-base neutralization reaction will occur, and the molecular chain will shrink until the original length is restored.

### 4.2. Hydrogels

In this section, stimulus response hydrogels, and their stimulus response mechanism, performance and devices are introduced in detail. In many deformed materials, hydrogels not only have good biological affinity, but also can be reversibly deformed in response to some stimuli [146,147]. The swelling degree of hydrogels depends on internal properties, including crosslinking density, microstructural anisotropy, and hydrophilicity. In particular, an important factor affecting the choice of manufacturing process and the final product is the printability of the hydrogel [148]. The advantage of using hydrogels is that they are biocompatible and easy to print using direct ink. For instance, when using the DIW or FDW method for printing, they require not only shear stress but also a certain yield strength. Hydrogel is an easy-to-synthesize material with high biocompatibility, adjustable, high alignment, low cost, etc. It is a promising interface material for biomedical applications, including non-invasive diagnosis, implantation therapy, cell manipulation, and implants [149].

#### 4.2.1. Thermally Responsive Hydrogels

**Figure 13:**
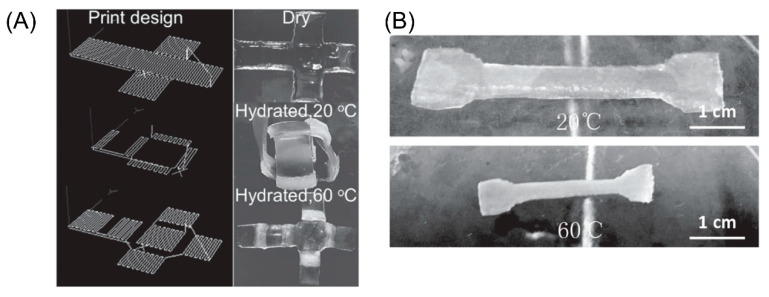
(**A**) A cubic box was formed by the printing pattern and through various temperature stimulations (Reproduced with permission from [155]). (**B**) 10% (*w/v*) N-isopropylacrylamide, an alginate/ Poly(N-isopropylacrylamide) (PNIPAAm) ionic covalent entanglement (ICE) hydrogel tensile specimen could swollen at different temperatures: 20 °C and 60 °C (Reproduced with permission from [154]).

Temperature responsive hydrogels refer to gels whose volume changes significantly when the ambient temperature changes. For temperature-sensitive hydrogels, due to the collapse or swelling behavior of the polymer chain itself at a critical temperature, the volume changes reversibly [150]. The most studied thermally responsive hydrogel material to date is Poly(N-isopropylacrylamide) (PNIPAm) [151,152,153]. This reversible temperature-related swelling of PNIPAm and its derivatives has been used in a variety of smart sensors and actuators. When the temperature of the aqueous solution is higher than its low critical solution temperature (LCST), the polymer network will fold, resulting in a smaller volume. Bakarich et al. designed a new type of hydrogel consisting of PNIPAm and alginate [154]. In this new type of composite hydrogel, combined with PNIPAm as a heat-sensitive material, alginate is used to enhance the mechanical properties. The team conducted a further study on the valve response speed, and showed that the microvalve could be opened and closed within 3.5 min, where the flow rate would decrease relative to the initial speed and remain stable later. Similarly, to produce shape deformed structures, Naficy et al. used PNIPAAm in combination with the thermally inactive polymer pHEMA [155]. Reversible transformation of shape can be achieved through heating and hydration. As shown in Figure 13B, there is a two-layer structure, consisting of PNIPAAm-based hydrogel at the top and PHEMA-based hydrogel at the bottom. Since the expansion rate of PNIPAAm is higher than that of PHEMA, when immersed in 20 °C water, the double-layer structure will bend to the PHEMA side. Figure 13A shows a cubic box made of the above double-layer structure. When the cube box is submerged in water and the temperature rises from 20 °C to 60 °C, it changes from the original cube structure to a flat structure. Of course, this process is also reversible.

#### 4.2.2. Light Responsive Hydrogels

**Figure 14:**
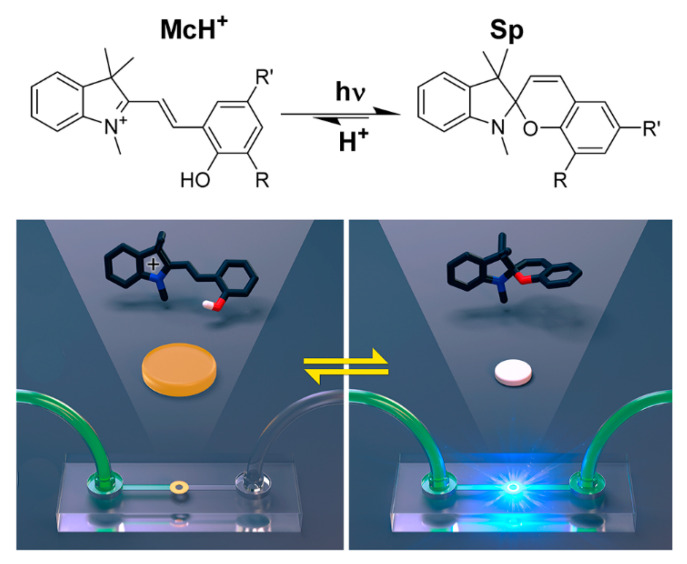
Molecular design of light-responsive hydrogels was fabricated for fast and reversible valves in a microfluidic device (Reproduced with permission from [156]).

Light responsive hydrogels differ from other responsive hydrogels, in that the light triggered responsive behavior can be controlled remotely without the need for the gel to be in direct contact with the environment. The light responsive hydrogels can provide the possibility of environmental stimulation based on the intensity of light or the directional illumination. Schiphorst et al., fabricated a reversible light-responsive hydrogel valves for a microfluidic device using spiropyran photoswitches and gel composition (Figure 14) [156]. These gels respond to light either through molecular exchange or through corresponding changes in physical or chemical properties such as viscosity, elasticity, shape, and swelling degree. Due to the high energy of short-wavelength light, many light responsive hydrogels have UV activity [157]. The chromophores act as molecular antennas in absorbing light on a hydrogel chain during exposure to light, causing chemical bonds to switch, break, or recombine within the hydrogel network [158].

#### 4.2.3. pH-Responsive Hydrogels

In this type of hydrogels, volume change mainly depends on the concentration of internal hydrogen ions in response to the change in pH [159,160,161]. Since the pH value of the human body varies greatly, from the strong acidity of the stomach, through the approximate neutrality of the blood and colon, to the weak acidity of the vagina, pH-responsive hydrogels are widely used in biomedicine.

**Figure 15:**
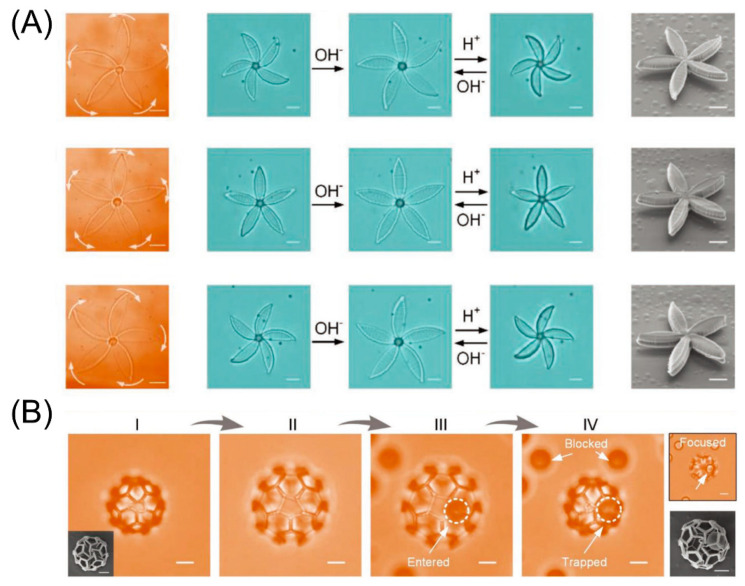
(**A**) Counterclockwise and clockwise twist of biomimetic micro-structures after contraction triggered by pH. (**B**) Functional microcages are prepared for selective capture and release of micro-objects by controlling the pore size (Reproduced with permission from [162]).

Hu et al. studied acrylic acid (AAc)-based hydrogels, placed them in alkaline and acidic solutions, and analyzed their swelling behavior in both states [162]. As shown in Figure 15A, when the pH value is greater than 9, the carboxyl group of AAc releases protons, causing its internal electrostatic repulsion to increase, which in turn causes the hydrogel to expand in volume. On the contrary, at a relatively low pH, its volume will shrink. Figure 15B shows the process of capturing microparticles through a series of pictures. When the structure is immersed in an alkaline solution (pH > 9), the cage-like hydrogel structure swells. At this time, the particles enter the cage with the flow of the liquid. In contrast, when it is placed in an acidic solution by changing the pH value of the solution (pH < 9), the cage-like hydrogel structure will shrink, trapping the particles in the cage successfully. Through continuous adjustment of the polymer system, it can finally adapt to the physiological pH of the human body, offering the possibility of subsequent potential applications in the field of medical engineering.

## 5. Applications of 4D Printing

### 5.1. Drug Delivery

**Figure 16:**
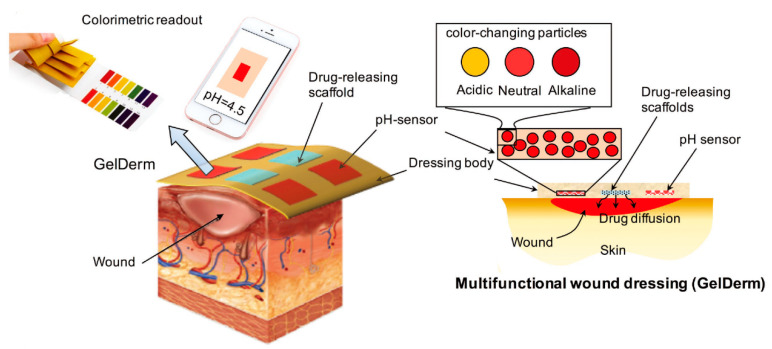
Schematic representation of an advanced multifunctional hydrogel-based dressing for treating epidermal wounds, with pH-sensitive and drug-eluting components. (Reproduced with permission from [167]).

In biomedical applications, hydrogels are widely used because they have good biocompatibility and flexibility [163]. However, how to precisely release the drug to the lesion site still remains a huge challenge. The so-called ideal drug delivery system refers to the system used to release drugs at the location of the disease through the changes of the environment and under controlled conditions. This is the ultimate goal that researchers need to achieve at present. Dai et al. proposed a new method, using a thermally responsive hydrogel (pluronic F127 diacrylic macromolecule) based shape memory hydrogel under the irradiation of near infrared light [164]. By adding graphene oxide, the composite material becomes photoresponsive. To fully restore the folded hydrogel to its original shape, it is only necessary to irradiate it for 240 s under near infrared light. The surface area caused by the shape change of the structure is different, which is the main factor affecting the drug release rate. Therefore, when the temporary shape is distorted, the surface area becomes smaller, and the drug release rate slower. Larush et al. used the DLP technology to create a drug release system in which the drug was released under the effect of pH and shape-dependent swelling [165]. In this study, they controlled the release of drugs by controlling the pH and surface area, demonstrating the ability of 3D printing technology to improve the performance of classic solid dosage forms. The ability of the oral drug delivery system to tailor the release of the drug depends mainly on the responsiveness of the printed object, and can use the change in the pH of the system to enable drug release at a specific location in the gastrointestinal tract. Taken together, these studies indicate that 4D printing provides the ability to manufacture structures that can control the localization and release rate of drugs [166]. As shown in Figure 16, Akbari et al. developed a directly activated drug delivery system based on 4D bioprinting [167]. First, they printed a set of porous sensors, mainly composed of alginate fibers and pH-responsive materials. At the same time, the drug-eluting stent was printed using alginate fibers loaded with gentamicin. The working process is as follows: The change of pH value can be captured by the sensor, and then the drug eluting stent can release the drug at the site where the pH value changes, so as to kill the bacteria.

With the development of smart materials that can respond to biological signals and pathological abnormalities in the body, the use of 4D bioprinting for drug delivery has become a reality. In order to manufacture a suitable drug delivery system, researchers must have a certain knowledge reserve in bioengineering technology, medicinal chemistry and pathophysiology. To promote wide applications in the clinic, researches must also be extensively conducted on safety and effectiveness.

### 5.2. Stents

The most basic function of a stent is to support the hollow structure. For instance, stents open arteries that have become narrowed or blocked because of coronary artery disease. In the past, after manufacturing, the stent needs to be transplanted into the patient’s body by surgery, which greatly increases the safety risk of the human body. Nowadays, with the advent of 4D bioprinting, stimuli-responsive materials smaller in size are being used to prepare scaffolds. After transplantation, with appropriate stimulation, the stent will automatically deform to a suitable size and shape, thus greatly lowering the risk of surgery.

So far, a large number of materials and 4D bioprinting methods available for manufacturing stents have been developed. Lonov et al. used 4D printing and hydrogel to make a self-folding stent with a hollow structure, with a minimum diameter of 20 μm [168]. When Ca2+ ion concentration changes, the polymer can undergo a reversible shape change. For stents made from such biocompatible hydrogels, The cell survival rate is relatively high. Ge et al. printed out a shape memory stents with high resolution for use in minimally invasive surgeries [73]. There are many technical requirements for the stent: First, the temporary shape of the stent can be maintained at a small diameter. Then, when the stent is transplanted into the blood vessel, the diameter of the blood vessel can be increased, and, when heated, the stent can return to its original shape. An adaptive structure was prepared by Liao et al. using 4D bioprinting. The structure is capable of self-expansion and self-shrinking under temperature changes.

**Figure 17:**
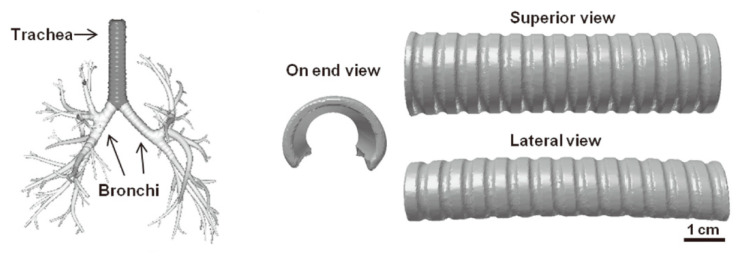
4D printing of shape memory-based personalized endoluminal tracheal stent (Reproduced with permission from [169]).

Most stents are used to treat vascular stenosis. However, with the development of technology, some new stents are being used in other intraluminal structures. Among them, the trachea is a more typical intraluminal structure. Due to the occurrence of disease, the trachea is narrowed or collapsed. As shown in Figure 17, the heat-driven lumen device, made by Cohn et al., uses shape-memory thermosetting materials [169]. As the temperature increases, the device can become a tracheal stent. Through custom design, the outline of the SMP structure can be reduced, thereby reducing the damage to the human body. 4D bioprinting has opened up a new path for manufacturing smart stents. In the future, the demand for 4D bioprinted stents in the medical field will increase. Better biocompatibility and better adaptation to human biological characteristics are still challenges in this regard.

### 5.3. Soft Robotics

**Figure 18:**
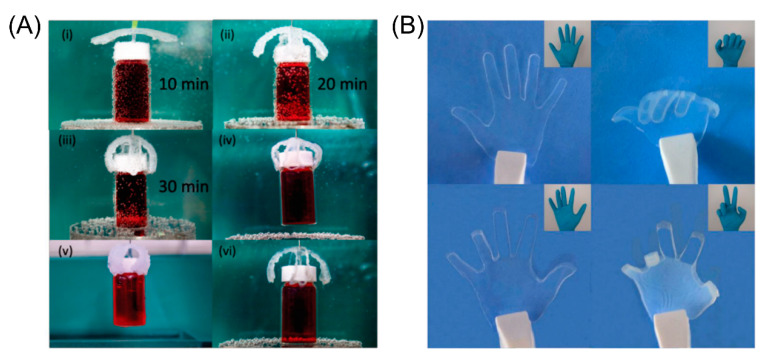
(**A**) 3D macroscopic gripper fabricated using shape-memory hydrogels at different bending positions (Reproduced with permission from [173]). (**B**) PAAm/PAAc hydrogel actuators were prepared with patterned photo-masks and actuated to play the game of“rock-paper-scissors” (Reproduced with permission from [174]).

The 4D printing technology not only has the flexible processing performance of complex structural parts, but also endows the material structure with unique intelligence and realizes the integration of structure and intelligence [170]. In recent years, many studies on soft robots made using 4D printing technology have been reported (Figure 18). The development of soft programmable materials, engineering design, and other scientific fields has also promoted the rapid development of soft robots. Compared with the traditional robots composed of motors, pistons, joints, and hinges, soft robots are more portable and flexible. They can flexibly change in size and shape according to actual needs, and can be added into more complex operations, with higher safety and environmental compatibility. Therefore, soft robots have a great application value and prospect in the medical and bionic fields. Yuk et al. prepared the grippers using hydrogels. The fish swimming in the water tank can be caught and released instantly by the grippers [171]. Kuo et al. researched and produced an intravascular gripper driven by a magnetic field. Palleau et al. used a sodium polyacrylate-based gel to make a gripper [172]. The special feature is that the operation is relatively gentle, and it can also operate on objects in the air.

More complex designs use various materials for controlled bending and twisting. Velders et al. demonstrated a technique that can perform controlled twisting and bending in the manner of human muscles [175]. The technique uses beads of sodium polyacrylate for simulation. Some researchers have developed a complex movement that can drive hydrogel software robots based on local circulation or oscillating chemical reactions [176].

### 5.4. Microfluidics

**Figure 19:**
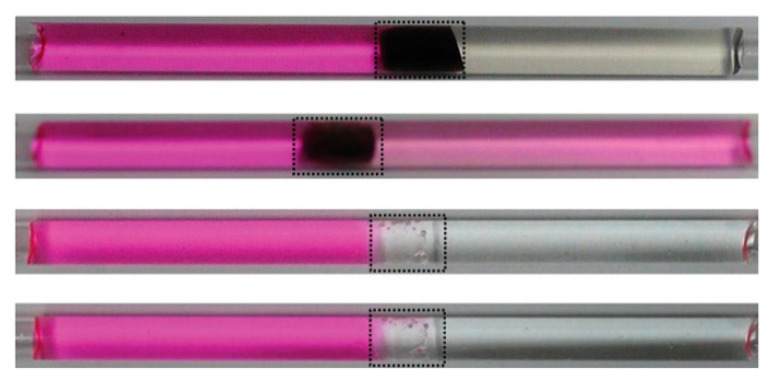
Liquid microvalves made with the poly(N-isopropylacrylamide) (PNIPAM)/ graphene oxide (GO) hydrogel can be controlled remotely using by a near-infrared(NIR) laser (Reproduced with permission from [181]).

Microfluidics have a wide range of applications in biomolecular and cell analysis, high-throughput screening, diagnosis, and treatment [177,178]. The operation and control of microfluidic devices need to be achieved with low power consumption, low cost, and unconstrained miniaturization. These requirements can be satisfied by some micro and smart components. Therefore, the integration of soft and stimulus-responsive hydrogels in microfluidic devices is a trend of future development. When there are external stimuli, it can accurately control the fluid through shape change. For example, D’eramo et al. designed microfluidic actuators using PNIPAm hydrogel columns at a horizontal resolution of 2 μm [179]. Up to 7800 microcages can be formed within 0.6 s by raising the system temperature of the hydrogel above LCST. Beebe et al. developed a smart hydrogel actuator by changing the pH to control microfluidics [180]. They utilized photolithography to fabricate different shapes of pH-responsive hydrogels, and the response time required was less than 10 s. Each hydrogel gate will respond to a specific pH value according to its chemical composition, and then automatically classify the fluid. As shown in Figure 19, Zhu et al. fabricated a liquid microvalve using this poly(N-isopropylacrylamide) (PNIPAM)/ graphene oxide (GO) nanocomposite hydrogel under control of an NIR laser [181].

### 5.5. Tissue Engineering

**Figure 20:**
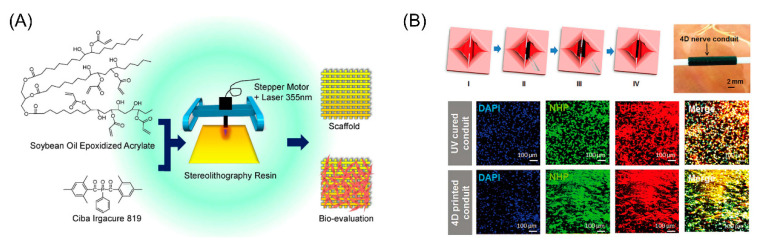
(**A**) 4D printing smart biomedical scaffolds with a novel soybean oil epoxidized acrylate (Reproduced with permission from [184]). (**B**) A reprogrammable nerve guidance conduit was designed and fabricated by stereolithographic 4D bioprinting for potentially repairing peripheral nerve injuries (Reproduced with permission from [68]).

With the rapid growth of the world’s population and the prominence of aging problems, higher demands are placed on medicine and on organs [182]. In order to create complex and dynamic tissues in vitro, 4D bioprinting needs to realize dynamic operations during the printing process. In recent research, the shape memory scaffolds have been proven to possess application potentials in minimally invasive delivery of functional tissues. The shape memory scaffolds can be integrated with bioelectronics and biodegradation equipment, and control the transmission process in a wireless and precise manner [183]. In addition, a major advance in the field of organ transplantation and tissue regeneration is the use of 4D bioprinting technology to implant stem cells directly into biological scaffolds. Miao et al. used the above method to cure a new type of renewable soybean oil epoxy acrylate on a biological scaffold to support the growth of bone marrow mesenchymal stem cells [184]. Experiments show that the bioscaffold has a higher ability of cell adhesion and proliferation, and can be completely restored to its original state at human body temperature. As shown in Figure 20B, Miao et al. demonstrated that a reprogrammable nerve guidance conduit was designed and fabricated by stereolithographic 4D bioprinting for potentially repairing peripheral nerve injuries. For tissue engineering in which living cells are involved, some stimulus (e.g., extreme pH value and high temperature) should be avoided. The application of 4D printing for tissue engineering is still in the stage of proof-of-concept study and this technique still has a long way for routine clinical application.

## 6. Conclusions and Prospective

4D printing (time as the fourth dimension) has been paid more attention to and spurred enormous interest since its emergence. Although this technique is mainly based on 3D printing and become an branch of additive manufacturing, the fabricated objects are no longer static and can be transformed into complex structures by changing the size, shape, property and functionality under external stimulation, which makes 3D printing alive. Due to the dynamic characteristic overcoming the limitations of traditional fabrication techniques, it has been widely applied to the field of soft robots, grippers, drug delivery, stent and tissue engineering. In this review, the AM technologies for 4D printing, implementation method of 4D printing, materials and application for 4D printing were presented.

AM technologies should be continuously improved to meet the requirement with high-resolution, high-speed and multi-materials fabrication. The resolution range of DIW and FFF is 100–200 μm which is limited by the diameter of the nozzle. Compared with vat photopolymerization methods, low printing speed is another limitation of extrusion-based methods due to the line-by-line printing mode. DLP as the most popular printing methods with high speed, can fabricate a layer at one time using the digital micromirror device to generate dynamic pattern. Furthermore, with design optical lens systems, DLP is able to fabricate structure at high resolution. On the other hand, the ability to print multiple materials simultaneously is future developing requirements of AM technologies. However, in general, FFF appears powerless and necessary prerequisites for DIW are using two compatible polymer resins. Inkjet printing seems easy to achieve multiple materials printing with multiple nozzles, but it will sacrifice the resolution. By designing switchable vats or modulating grayscale light, DLP techniques would produce structures with multiple materials. A combination of different AM technologies is another trend of 4D printing. It was noticed that these technologies and equipment have their own special features and we should combine different techniques with absorbing the advantage of different technologies.

As one of the most critical challenges facing 4D printing, novel smart materials should be developed for further expansion of 4D printing. Although various type of materials including shape memory polymers and stimulus response hydrogels has been successfully investigated for 4D printing, slow response rate and low efficiency hamper further development. For instance, Song et al. deposited the graded water-responsive elastomer materials onto a heat-shrinkable shape memory polymer to form dual-stimuli self-morphing structures. However, the whole time of completely deformation under the stimulus is nearly 20 min. Chen et al. designed a high-performance integrated sensor–actuator with strain-sensing and temperature self-sensing and the average materials response time is about 20 s [185]. On the other hand, most existing materials make the response to only one stimulus and it will not work in the case of stimulus producing equipment failure. Therefore, materials which are responsive to multi- stimulus are needed to be developed. We believe that future combinations of various types of responsive materials will bring outstanding breakthroughs to 4D printing. In addition, for 4D bioprinting, the good biocompatibility and proper mechanical properties are the essential requirements for ideal materials. So far most bio-materials currently used feature good biological compatibility, but the mechanical property has not yet been tested.

Although a variety of stimuli-responsive microstructures have been reported, the embryonic 4D printing needs a significant amount of efforts including the development and improvement of new materials and printing methods. Despite the outlined challenges similar to other emerging technologies, 4D will certainly to have a big impact and good prospects for practical applications in the near future.

## Figures and Tables

**Table 1:** Stimulation method of four-dimensional 4D printing.

Stimulation Method	Theory	Advantages	Disadvantages
Water/humidity	Swelling/Shrinkage	Clean/Convenient	Slow response [80,81]
Temperature	Internal stress inequality	Controlled adjustable	Slow response, complicated [82,83,84]
Light	Photo-thermal effect	High-resolution control//remote control	Complicated [85,86,87,88]
Electric field	Electro-thermal effect	Fast	Operating inconvenience [89,90]
Magnetic field	Magnetic drive	Remote control	Operating inconvenience [91,92,93]
Cell traction force (CTF)	Actin binding and interaction.	Biological compatibility	Cell traction force small and hard to control, high design requirement [94,95]
